# Hypermethylated *14-3-3-σ *and *ESR1 *gene promoters in serum as candidate biomarkers for the diagnosis and treatment efficacy of breast cancer metastasis

**DOI:** 10.1186/1471-2407-10-217

**Published:** 2010-05-20

**Authors:** Mercedes Zurita, Pedro C Lara, Rosario del Moral, Blanca Torres, José Luis Linares-Fernández, Sandra Ríos Arrabal, Joaquina Martínez-Galán, Francisco Javier Oliver, José Mariano Ruiz de Almodóvar

**Affiliations:** 1 Center for Biomedical Research and Institute of Biopathology and Regenerative Medicine, Granada University, Granada, Spain; 2 Radiation Oncology, Hospital Virgen de las Nieves, Granada, Spain; 3 Instituto Canario de Investigación del Cáncer and Servicio de Oncología Radioterápica, Hospital Dr. Negrín, Gran Canaria, Spain; 4 CIBER de Epidemiología y Salud Pública, Hospital Universitario San Cecilio, Granada, Spain; 5 Instituto de Parasitología y Biomedicina, López-Neira, CSIC, Granada, Spain; 6 Hospital Universitario San Cecilio, Granada, Spain

## Abstract

**Background:**

Numerous hypermethylated genes have been reported in breast cancer, and the silencing of these genes plays an important role in carcinogenesis, tumor progression and diagnosis. These hypermethylated promoters are very rarely found in normal breast. It has been suggested that aberrant hypermethylation may be useful as a biomarker, with implications for breast cancer etiology, diagnosis, and management. The relationship between primary neoplasm and metastasis remains largely unknown. There has been no comprehensive comparative study on the clinical usefulness of tumor-associated methylated DNA biomarkers in primary breast carcinoma and metastatic breast carcinoma. The objective of the present study was to investigate the association between clinical extension of breast cancer and methylation status of *Estrogen Receptor1 *(*ESR1*) and *Stratifin *(*14-3-3-σ*) gene promoters in disease-free and metastatic breast cancer patients.

**Methods:**

We studied two cohorts of patients: 77 patients treated for breast cancer with no signs of disease, and 34 patients with metastatic breast cancer. DNA was obtained from serum samples, and promoter methylation status was determined by using DNA bisulfite modification and quantitative methylation-specific PCR.

**Results:**

Serum levels of methylated gene promoter *14-3-3-σ *significantly differed between Control and Metastatic Breast Cancer groups (P < 0.001), and between Disease-Free and Metastatic Breast Cancer groups (P < 0.001). The ratio of the *14-3-3-σ *level before the first chemotherapy cycle to the level just before administration of the second chemotherapy cycle was defined as the Biomarker Response Ratio [BRR]. We calculated BRR values for the "continuous decline" and "rise-and-fall" groups. Subsequent ROC analysis showed a sensitivity of 75% (95% CI: 47.6 - 86.7) and a specificity of 66.7% (95% CI: 41.0 - 86.7) to discriminate between the groups for a cut-off level of BRR = 2.39. The area under the ROC curve (Z = 0.804 ± 0.074) indicates that this test is a good approach to post-treatment prognosis.

**Conclusions:**

The relationship of *14-3-3-σ *with breast cancer metastasis and progression found in this study suggests a possible application of *14-3-3-σ *as a biomarker to screen for metastasis and to follow up patients treated for metastatic breast cancer, monitoring their disease status and treatment response.

## Background

Breast cancer is a major health problem, with more than 1,000,000 new cases and 370,000 deaths annually worldwide. Over the past decade, breast cancer mortality has been declining in the majority of developed countries, despite an increasing incidence. This is the combined result of better education, widespread screening programs, and more efficacious adjuvant treatments. Furthermore, improved knowledge of breast cancer biology now allows the majority of breast cancer patients to be spared the cosmetic, physical, and psychological consequences of radical treatment, including radiotherapy [1-3].

However, clinicians have limited instruments available for the early detection of breast cancer recurrence that results from metastases undetected at the time of the primary treatment. Molecular studies have yielded important data on breast cancer development and progression. However, no biomarkers, used alone or together, have yet proved able to definitively predict the outcome of cancer treatment, and new molecules are required that can serve as reliable indicators of the risk of cancer recurrence. Serum biomarkers are produced by body organs or tumors, and large amounts in the blood can be suggestive of tumor activity [4]. The only breast cancer tumor markers with demonstrated clinical utility are tumor-associated antigens, but their usefulness to follow up patients with metastatic disease can be limited [5].

A key challenge in breast cancer therapy is to elucidate the mechanisms involved in inducing or repressing the multiple genes required for cancer cell growth, invasion, and metastasis. Methylation-associated changes affect numerous genes in all cellular pathways [6], and it is widely accepted that a succession of accumulative hits in oncogenes lead to genetic lesions. The pathological features of breast cancer follow a sequential progression from the transition of a normal cell to benign proliferative hyperplasia, hyperplasia with atypia, carcinoma *in situ*, and, eventually, invasive and metastatic disease [7]. However, the timetable of epigenetic alterations during this progression is little understood [8]. Some studies have evaluated the association between gene hypermethylation and biological or clinical properties of breast tumors [9-12].

In breast cancer, tumor-related genes may be silenced by hypermethylation. DNA methylation, unlike other epigenetic changes, does not alter the nucleotide sequence. Hypermethylation is an epigenetic change that blocks the promoter region of a gene and results in gene silencing. When CpG islands are hypermethylated, the activity of the regulatory proteins that promote transcription is restricted due to the tightly packed nucleosomes[13]. Many hypermethylated genes have been reported, and silencing of these genes plays an important role in carcinogenesis, tumor progression [9,14], and diagnosis [11,15-17]. These hypermethylated promoters are very rarely found in normal breast. It has been suggested that aberrant hypermethylation may be useful as a biomarker, with implications for breast cancer etiology, diagnosis, and management.

The epigenetic alterations that initiate and drive tumorigenesis are promising targets for the early detection of tumor and perhaps metastasis, because they may precede clinical signs of cancer or recurrence and can be detected at very low levels [11]. The relationship between primary neoplasm and metastasis remains largely unknown [18]. There has been no comprehensive comparative study on the clinical usefulness of tumor-associated methylated DNA biomarkers in primary breast carcinoma and metastatic breast carcinoma.

Although numerous issues remain to be resolved, the quantitative measurement of circulating methylated DNA remains a promising approach to cancer risk assessment. The objective of the present study was to assess the usefulness of serum concentrations of methylated *Estrogen Receptor1 (ESR1) *and *Stratifin *(*14-3-3-σ) *gene promoters in breast cancer patients in two very different clinical situations: i) treated and with no evidence of residual or recurrent disease, and ii) treated and with detected metastatic breast cancer. We also examined whether these biomarkers add information of clinical utility during the post-treatment follow-up of breast cancer patients with metastases.

## Methods

### Samples

Blood samples (7 ml) were obtained from all study participants by venipuncture. All samples were randomly coded before processing to ensure that analysts were blinded to their origin. Samples were centrifuged at 2000 g for 10 min at room temperature, and 1-ml aliquots of serum samples were carefully transferred into new tubes. Sera were stored at -80°C until their analysis (between June 2008 and March 2009). Full clinical and pathological data were collected and known for all participants. Patient characteristics are summarized in Table 1. This research was approved by the Institutional Ethics Committees of the Negrín Hospital, Gran Canaria and the Virgen de las Nieves Hospital, and the University of Granada, Spain, and written informed consent was obtained from all study participants.

**Table 1:** Clinical and pathological characteristic of the breast cancer patients

Characteristics	Disease-Free Breast Cancer Group	Metastatic Breast Cancer Group
**Histological type**		
Invasive ductal carcinoma	63 (81.8%)	22 (64.7%)
Invasive lobular carcinoma	8 (10.4%)	6 (17.6%)
Other invasive carcinoma	6 (7.8%)	4 (11.4%)
Unknown	0	2 (5.8%)

**Histological grade**		
Grade I	17 (22.1%)	1 (2.9%)
Grade II	25 (32.5%)	8 (23.5%)
Grade III	26 (33.8%)	20 (58.8%)
Unknown	9 (11.7%)	5 (14.7%)

**Tumor size**		
Tis	2 (2.6%)	1 (2.9%)
T1	40 (52.0%)	10 (29.4%)
T2	24 (31.2%)	13 (38.2%)
T3	7 (9.1%)	5 (14.7%)
T4	4 (5.2%)	2 (5.8%)
Tx	0	1 (2.9%)
Unknown		2 (5.8%)

**Node involvement**		
N0	41 (53.3%)	13 (38.2%)
N1	26 (33.8%)	8 (23.5%)
N2	6 (7.8%)	9 (26.4%)
N3	3 (3.9%)	2 (5.8%)
Nx	1 (1.3%)	2 (5.8%)

**Estrogen receptor status**		
Negative	20 (25.1%)	10 (27.7%)
Positive	53 (68.8%)	21 (61.7%)
Unknown	4 (5.2%)	3 (8.8%)

**Progesterone receptor status**		
Negative	23 (30.0%)	10 (29.4%)
Positive	50 (64.9%)	22 (64.7%)
Unknown	4 (5.1%)	2 (5.8%)

**Menopause**		
Yes	46 (62.3%)	31 (91.1%)
No	26 (33.8%)	
Unknown	3 (3.9%)	3 (8.8%)

Patient groups were formed as follows:

a) Disease-Free Breast Cancer group [DFBC]. A group of 77 consecutive women surgically treated at Hospital Negrín, Gran Canaria, Spain for localized operable breast cancer without clinical or radiological evidence of distant metastases were enrolled in this study between May 2007 and December 2008.

b) Metastatic Breast Cancer Group [MBC]. Samples were obtained from 34 consecutive patients with metastatic breast cancer disease treated in the Virgen de las Nieves Hospital, Granada, Spain. Samples were taken on the day that each chemotherapy cycle started, gathering a series of sequential samples for each patient from the first to the last CT cycle.

c) Healthy Control Group, [HC]. An age-matched sampling approach (with FDBC group) was used, obtaining blood samples from 34 women randomly selected from among healthcare professionals of our hospital undergoing regular health examinations at the Department of Preventive Medicine.

### DNA isolation

DNA from serum samples (2 ml per column) was obtained by using QIAmp DNA Blood Kit (QIAGEN Inc., CA) according to manufacturer's recommendations. A final elution volume of 200 μl was established. Extracted DNA was quantified spectrophotometrically. The amount of DNA recovered was measured as μg/sample. DNA samples were stored at -80°C until use.

### DNA bisulfite modification and real-time QMS-PCR using SYBR green

Identical DNA sequences that differ only in methylation status [19] can be amplified by means of Quantitative Methylation Specific PCR (QMS-PCR). Reagents required for the bisulfite modification of DNA were supplied in the CpGenomeTM DNA Modification Kit (Intergen, MA). The process was performed according to manufacturer's recommendations. Sufficient DNA can be recovered to perform MSP from an amount of starting material as small as 0.001 μg. In brief, 100 μl of extracted DNA was treated with sodium bisulfite for 16 h, thereby converting all unmethylated cytosines to uracils but leaving methylcytosines unaltered. Efficiency of DNA recovery after bisulfite modification was around 55% (data not shown). One microliter of the recovered bisulfite-treated DNA was used in each well for SYBR green reaction. Modified DNA of standards and samples are stable for at least 2 months at -80°C. A sample of bisulfite-modified universally methylated DNA genome (CpGenomeTM Universal Methylated DNA, Intergen, New York, USA), treated in the same way as patient samples and adjusted after modification to 2 μg/ml (quantified spectrophotometrically), served as internal standard to prepare serial dilutions (from 1 to 1/128) with MiliQ water to construct a Standard Curve for Real-Time QMS-PCR. Each plate contained patient samples, serial dilutions of completely methylated DNA for constructing calibration curves, positive controls, and two wells with water blanks used as negative controls. In all cases, correlation coefficients for the calibration curves were higher than 0.98, slopes ranged from 3.2 to 3.4, and PCR efficiencies were around 100%.

The reaction mixture contained 1 μl of modified serum DNA of each standard or unknown sample as template for real-time QMS-PCR, 0.5 μM of each oligonucleotide primer, 12.5 μl of 2× SYBR Green Supermix (Bio-rad), and sterile water. All PCR experiments were performed in a volume of 25 μl with 96-well plates. Primer sequences were obtained from previously published data for *stratifin *(*14-3-3-σ*) [20] and *estrogen receptor-*α (*ESR1*) [21]. The fluorescence signal of the quantitative methylation-specific PCR was generated by SYBR Green Super Mix (BioRad, Hercules, CA). PCR amplification was done by using a previously reported procedure [11]. The fluorescence value after QMS-PCR in each sample was converted into units of universally methylated DNA (μg/ml), which we designated *"relative units"*, using the corresponding PCR Standard Curve obtained from the iCycler iQ software. Results obtained in a previous study [11] indicated that the method was valid for this investigation.

### Statistical analysis

The associations of the two biomarkers with breast cancer presence, their capacity to discriminate between women with and without clinical and radiological evidence of breast cancer metastasis, and their post-chemotherapy behavior were analyzed in the following phases:

(1) Descriptive analysis of the three groups (DFBC, MBC, and HC) was performed for each biomarker, expressing results as means and standard error of the means (SEM).

(2) The Kruskal-Wallis test was used to study differences among groups (DFBC, MBC, and HC), and the Dunn's multiple comparison test was used for paired comparisons when results were significant.

(3) Effects of the chemotherapy on serum *ESR1 *and *14-3-3-σ *levels were studied using Wilcoxon signed rank test and paired test.

(4) Effects of the chemotherapy on treated patients with detected metastatic breast cancer were measured according to the following scoring system: Measurable Response. (MR): disappearance or decrease of all signs and symptoms of the lesions, no growth of any lesion, and no appearance of a new lesion. *Stable Disease *(SD): no significant changes in lesion size or in any tumor-related signs or symptoms. *Progression *(P): measurable increase in lesion size or appearance of new lesions. *Mortality during treatment *(MDT): patients dying during the chemotherapy period.

(5) Biomarker Response Ratio (BRR). The ratio of the *14-3-3-σ *level before the first chemotherapy cycle to the level just before administration of the second chemotherapy cycle was defined as the BBR.

(6) ROC curves: The distributions of the *14-3-3σ *ratio values corresponding to different patient populations are, at least in part, overlapped. As a consequence, the number of correct predictions of the measurable response (true positive (TP) cases identified by means of the test depends on the threshold level selected. Using different thresholds, it is possible to obtain successive pairs of false positive (FP) and TP values that can be plotted as a curve. A useful numerical parameter arising from this graph is the proportion of ROC space that lies below the ROC curve (Z).

## Results

Table 1 shows the general characteristics of the two patient groups studied. The groups did not differ in histological type (P > 0.05), estrogen receptor status (P > 0.05) or iii) progesterone receptor status (P > 0.05). As expected, they significantly differed in tumor size (P < 0.05), nodal involvement (P < 0.05), and histological grade (P < 0.05).

**Figure 1:**
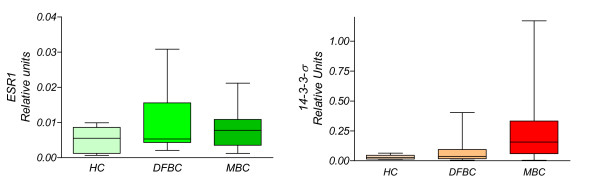
**The box and whisker plot shows the median value and 10-90 percentiles of biomarkers, *ESR1 *and *14-3-3-σ*, measured in the serum of the individuals in each of the three study groups: Healthy Controls (*HC*); Disease-Free Breast Cancer *(DFBC)*; and Metastatic Breast Cancer *(MBC)***. *14-3-3-σ *values significantly differed between the *DFBC *and *MBC *groups (Dunn test, P > 0.0001) and between each of these and the HC group (P < 0.001).

Median serum levels of methylated gene promoter *ESR1 *did not differ among HC, DFBC, and MBC groups (P > 0.05). Median serum *14-3-3-σ *values did not differ between HC and DFBC groups (P > 0.05), but differed between HC and MBC groups (P < 0.001) and between DFBC and MBC groups (P < 0.001). Figure 1 depicts the results obtained.

Calculation of the area under the ROC curve *(Z) *for the capacity of *14-3-3-σ *to discriminate between healthy individuals and patients with breast cancer metastatic disease gave a value of *Z *= 0.925 (95% confidence interval: 0.886 to 0.964), an excellent level of accuracy.

**Figure 2:**
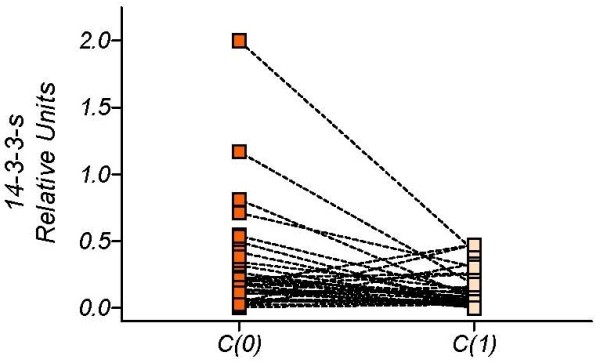
***14-3-3-σ *values measured in serum of patients with metastatic breast cancer disease before and after the first chemotherapy cycle**. Wilcoxon signed rank test, P = 0.0045.

Figure 2 depicts *14-3-3-σ *gene values before and after the first chemotherapy cycle, showing that the chemotherapy produced a major reduction in the serum levels of *14-3-3-σ *in the MBC group. These differences have been studied using Wilcoxon signed rank test and show that the median difference in methylation after treatment is greater than zero (P = 0.0045); being the paired test also significant (P = 0.012).

**Figure 3:**
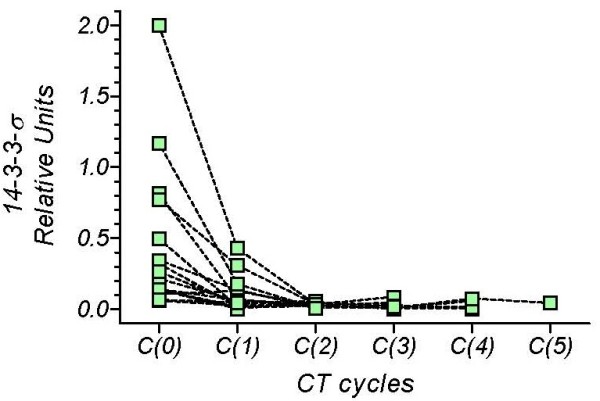
***14-3-3-σ *values measured in serum of patients with breast cancer metastatic disease before the first C(0) and successive C(1...5) chemotherapy cycles, in patients with "continuous decline" biomarker pattern**.

**Figure 4:**
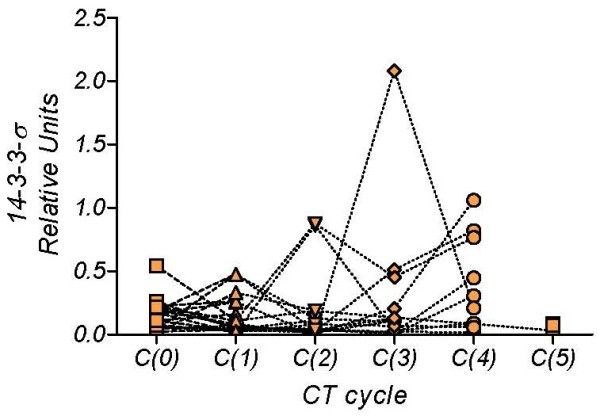
***14-3-3-σ *values measured in serum of patients with breast cancer metastatic disease before the first C(0) and successive C(1...5) chemotherapy cycles, in patients with "rise-and-fall" biomarker pattern**.

However, although initial levels appear to fall in most of the MBC patients, other patients show no change or an increase in levels. Figure 3 shows those MBC patients with a continuous decline in serum *14-3-3-σ*, and Figure 4 those patients with both rises and falls. This biomarker-based categorization has been empirically defined. Table 2 shows the contingency table that was constructed by combining this biomarker's response-pattern with the chemotherapy response scores. Analysis with the Pearson chi-square test gave a value of 10.23 (P = 0.017), indicating that the time course of the biomarker was determined by the clinical response to the treatment. In summary, the continuous-decline pattern of serum *14-3-3-σ *levels was associated with a positive predictive value of 65% (95% CI 38-86%), implying a favorable prognosis in two out of three patients, whereas the rise-and-fall pattern was associated with a negative predictive value of 88%, implying a poor prognosis for most of the patients with this pattern.

**Table 2:** Patients' summary score distribution (treatment response distribution) according to the biomarker pattern observed during the treatment time-course.

Pattern	MR	SD	P	MDT
Continuously decline	11	2	3	1

Rise and fall	2	6	6	3

**MR: **Measurable Response; **SD: **Stable Disease; **P: **Progression; **MDT: **Mortality during treatment

**Figure 5:**
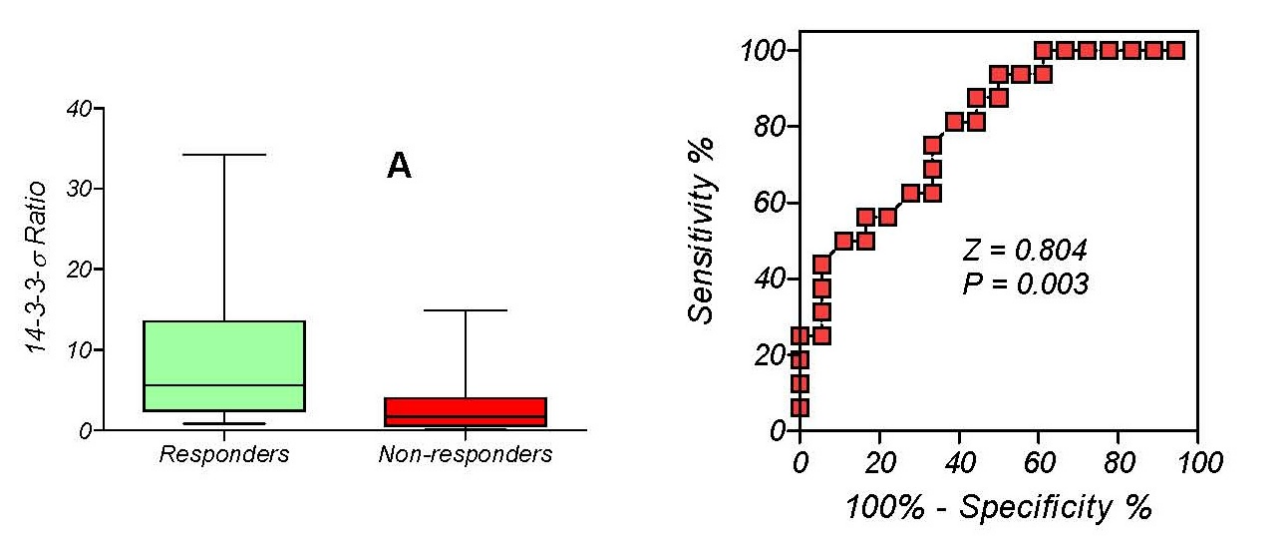
**Discriminatory power of the biomarker response ratio [*14-3-3-σ BRR*] to predict the outcome in patients with metastatic breast cancer treated with chemotherapy**. A: Comparison between treatment outcomes; B: ROC curve.

Finally, we calculated the ratio of the *14-3-3-σ *level before the first chemotherapy cycle to the level just before administration of the second chemotherapy cycle for the "continuous decline" and "rise-and-fall" groups (figure 5). The median values are: 5.606 and 1.694, respectively and the medians, Mann Whitney test, are significantly different (P = 0.0034).

## Discussion

Accurate prognosis and predictive factors are necessary for the optimum management of patients with cancer and are especially important in breast cancer, because of its widely varying outcomes and the availability of potentially beneficial systemic adjuvant therapies. The definitive assessment of the clinical value of a predictive factor is a long process [4], but the use of an unbiased genome-wide approach has permitted the rapid identification of a number of genes that strongly predict a poor clinical outcome [22]. The identification of mutations and/or epigenetic alterations in cancer may be useful to develop novel, more effective biomarkers and therapies in breast and colon cancer [22]. In breast cancer, promoter hypermethylation has been reported for various genes that cover most cell functions [11,15,17,23,24].

Estrogen receptor status is an important factor in the diagnosis and prognosis of breast cancer. A previous study by our group found a significant difference in serum values of *ESR1 *and *14-3-3-σ *gene promoters between breast cancer patients and healthy controls [11]. The present study also found a significant difference in serum methylated *14-3-3-σ *gene promoter between metastatic breast cancer patients and healthy controls. In contrast, however, *ESR1 *appeared to be unmethylated in the present patients with metastatic breast cancer. Theoretically, *ESR1 *is considered to be preferentially methylated in tumors because its inactivation confers a selective clonal advantage[25]. It is possible that the specific environmental and nutritional setting of breast cancer metastases produces changes in this epigenetic alteration. However, further in-depth study is required to explain this intriguing finding. Reports of differences in methylation pattern between primary and metastatic breast cancer [18] may indicate that therapeutic targets in primary breast cancer are not be the same as targets in metastatic sites.

14-3-3 proteins are crucial in a wide variety of cell responses, including DNA damage checkpoints and apoptosis [26]. Disruption of the G2-M checkpoint also appears to contribute to the change in the sensitivity of cells to chemo- and radiotherapy [27,28]. 14-3-3 sigma sequesters the cdc2-cyclin B1 complex in the cytoplasm, resulting in G2 arrest. Among the genes involved in the G2-M checkpoint, *14-3-3σ*, a transcriptional target of p53, is frequently silenced by DNA methylation of the *14-3-3σ *gene promoter or by induction of estrogen-responsive ubiquitin ligase that specifically targets *14-3-3σ *for proteosomal degradation [23,29]. The inactivation and reduced expression of 14-3-3σ have been reported in various cancers, including breast cancer [16,26,30]. To date, the sigma isoform of 14-3-3 proteins has been the isoform most directly implicated in carcinogenesis and is recognized as a tumor-suppressor gene [31]. Although the molecular basis for the tumor-suppressor function of 14-3-3σ is unknown [26], it has been suggested that 14-3-3σ is a critical regulator of G2-M [32]. It has also been demonstrated that endogenous 14-3-3σ preferentially forms homodimers in cells [33]. Knocking out *14-3-3σ *in cancer cells leads to mitotic catastrophe and cell death from DNA damage due to the absence of G2-M arrest [34]. Moreover, the highly conserved human *14-3-3 *gene family encodes proteins with either tumor-promoting or tumor-suppressing activities, suggesting that the cellular balance among different 14-3-3 isoforms is crucial for the proper functioning of cells [32]. 14-3-3 proteins have been found in primary breast cancer, enhancing its biological activity [35]. The structure of the p53 C-terminus bound to the adaptor protein 14-3-3 has been recently described, providing a rationale for the observed stabilizing effect of 14-3-3 binding [36]. Consistent with these data, the G2-M checkpoint is impaired in cancer cell lines that show methylation of *14-3-3 σ*, while restoration of the expression of these genes using 5-aza-dC restores G2-M arrest induced by DNA damage [37]. This molecule also contributes to mitotic catastrophe in carcinoma cells treated with chemotherapy agents [38].

Results of a recent study [39] showed that 14-3-3 and HSP70 expression may be useful as biomarkers and targets for the diagnosis and treatment of human triple-negative breast cancer. Breast cancer metastasis is the main cause of treatment failure, and the goal of adjuvant therapy is to eliminate disseminated tumor cells after complete removal of the tumor. However no tool is available to monitor its efficacy [11]. Response to adjuvant treatment is usually evaluated retrospectively based on recurrence and survival rates. Therefore, the identification of metastasis biomarkers at an early stage may contribute to the early diagnosis and treatment of breast cancer patients. There is increasing recognition of the importance of epigenetic changes in the metastatic process. Cells may acquire an epi-genotype that allows them to disseminate from the primary tumor mass or survive and proliferate at a secondary tissue site [40]. Overall, these results offer evidence of a difference in protein profile between metastatic and primary breast cancer.

The expression profile of the metastatic tumor is known to differ between primary tumor and heterogeneous metastasis [39]. The present findings show that breast cancer methylation profiling might yield biomarkers for the diagnosis and treatment efficacy of breast cancer metastasis. Thus, we found that ROC analysis of serum levels of *14-3-3-σ *methylated gene-promoter discriminated between healthy individuals and metastatic breast cancer patients with a sensitivity of 81% (95% CI: 74.0 - 86.8) and a specificity of 96.2% (95% CI: 80.45 - 99.9), making this biomarker a candidate for use in metastasis screening in the follow-up of treated breast cancer patients. It would be of special interest to investigate whether the elevated post-surgical serum *14-3-3-σ *levels in some of the present patients (DFBC group) and in our previous study [11] indicate a risk of metastatic cancer or tumor recurrence. Although this type of investigation requires a prolonged follow-up [4], identification of this biomarker as a risk factor and its correlation with other clinical factors may lead to improvements in breast cancer treatment.

14-3-3-σ proteins are known to be crucial in a wide variety of cell responses, including cell cycle progression, DNA damage checkpoints, and apoptosis [26], and *14-3-3-σ *hypermethylation is a significant event in primary breast cancer [41]. However, its impact on tumor progression and its potential as a predictive factor remain unknown. Because 14-3-3-σ proteins regulate normal cell processes, the loss of their expression (mainly by hypermethylation of *14-3-3-σ *gene promoter) may be implicated in breast cancer progression [31,42]. This hypothesis is supported by our data, since hypermethylation of *14-3-3-σ *was significantly associated with the response to metastatic breast cancer treatment. We calculated BRR values for the "continuous decline" and "rise-and-fall" groups (values plotted in Figure 5A). Analysis by unpaired t-test with Welch's correction showed a significant difference in mean values between these groups (P = 0.021). Subsequent ROC analysis, considering the "continuous decline group" as controls and the rise-and-fall group as cases (Figure 5B), showed a sensitivity of 75% (95% CI: 47.6 - 86.7) and a specificity of 66.7% (95% CI: 41.0 - 86.7) to discriminate between the groups for a cut-off level of BRR = 2.39.

The area under the ROC curve (Z = 0.804 ± 0.074) indicates that this test is a good approach to post-treatment prognosis and supports the very recent idea that 14-3-3 proteins may be related to breast cancer metastasis and evolution [43]. However, a small amount of *14-3-3-σ *methylated was detected in sera from healthy controls [11], which may be explained by presence of occult benign breast disease, although other possible sources of this DNA include: normal tissues, which show higher methylation values with increasing age[44]; leukocytes [23], or breast benign disease [41]. The above findings raise some questions about the utilization of *14-3-3-σ *gene promoter in DNA extracted from serum for metastasis screening in the follow-up of treated breast cancer patients. Further research is warranted to elucidate this issue and to establish the impact of *14-3-3σ *on tumor progression and its potential to predict the response to treatment of metastatic breast cancer.

## Conclusions

There are numerous promising treatments for advanced breast cancer now in phase III trial, and there is an urgent need to establish sensitive end-points that can be assessed earlier than overall survival [45]. Although further research is required to establish the link between *14-3-3-σ *hypermethylated gene promoter measured in the serum of breast cancer patients and the response to chemotherapy, including control of symptoms, avoidance of adverse effects, and improvement in quality of life, important aspects in the approaching era of personalized medicine, this biomarker may be potentially useful to monitor disease status and treatment response.

## Abbreviations

ESR1: Estrogen Receptor1; 14-3-3-σ: Stratifin; DFBC: Disease-Free Breast Cancer group; MBC: Metastatic Breast Cancer Group; HC: Healthy Control Group; QMS - PCR: Quantitative methylation-specific - polymerase chain reaction; ROC curve: Receiver operating characteristic curve; BBR: Biomarker Response Ratio;

## Competing interests

The authors declare that they have no competing interests.

## Authors' contributions

MZ, JMG, PL and RdM were significantly involved in patient recruitment and patient treatment contributed to the correlation of clinical data with experimental findings. PL and RdM took a role in supervising the final version of the article, BT, JLL-F and SRA carried out the in vitro analysis, FJO take a role in the interpretation and discussion of results; JMRdA conceived and designed the study, interpreted the data, and revised the paper giving final approval of the version submitted. All the authors read and approval the final manuscript.

## Pre-publication history

The pre-publication history for this paper can be accessed here:

http://www.biomedcentral.com/1471-2407/10/217/prepub

## References

[B1] Guarneri V Conte PF The curability of breast cancer and the treatment of advanced disease Eur J Nucl Med Mol Imaging 2004 31 Suppl 1 S149 161 10.1007/s00259-004-1538-5 15107948

[B2] Lopez E Nunez MI Guerrero MR del Moral R de Dios Luna J del Mar Rodriguez M Valenzuela MT Villalobos M Ruiz de Almodovar JM Breast cancer acute radiotherapy morbidity evaluated by different scoring systems Breast Cancer Res Treat 2002 73 2 127 134 10.1023/A:1015296607061 12088115

[B3] Pinar B Lara PC Lloret M Bordon E Nunez MI Villalobos M Guerrero R Luna JD Ruiz de Almodovar JM Radiation-induced DNA damage as a predictor of long-term toxicity in locally advanced breast cancer patients treated with high-dose hyperfractionated radical radiotherapy Radiat Res 2007 168 4 415 422 10.1667/RR0746.1 17903032

[B4] Ruiz-Garcia J Ruiz de Almodovar JM Olea N Pedraza V Thyroglobulin level as a predictive factor of tumoral recurrence in differentiated thyroid cancer J Nucl Med 1991 32 3 395 398 2005446

[B5] Stearns V Yamauchi H Hayes DF Circulating tumor markers in breast cancer: accepted utilities and novel prospects Breast Cancer Res Treat 1998 52 1-3 239 259 10.1023/A:1006137619153 10066086

[B6] Esteller M Dormant hypermethylated tumour suppressor genes: questions and answers J Pathol 2005 205 2 172 180 10.1002/path.1707 15643671

[B7] Beckmann MW Niederacher D Schnurch HG Gusterson BA Bender HG Multistep carcinogenesis of breast cancer and tumour heterogeneity J Mol Med 1997 75 6 429 439 10.1007/s001090050128 9231883

[B8] Fraga MF Herranz M Espada J Ballestar E Paz MF Ropero S Erkek E Bozdogan O Peinado H Niveleau A Mao JH Balmain A Cano A Esteller M A mouse skin multistage carcinogenesis model reflects the aberrant DNA methylation patterns of human tumors Cancer Res 2004 64 16 5527 5534 10.1158/0008-5472.CAN-03-4061 15313885

[B9] Bae YK Brown A Garrett E Bornman D Fackler MJ Sukumar S Herman JG Gabrielson E Hypermethylation in histologically distinct classes of breast cancer Clin Cancer Res 2004 10 18 Pt 1 5998 6005 10.1158/1078-0432.CCR-04-0667 15447983

[B10] Garcia JM Silva J Pena C Garcia V Rodriguez R Cruz MA Cantos B Provencio M Espana P Bonilla F Promoter methylation of the PTEN gene is a common molecular change in breast cancer Genes Chromosomes Cancer 2004 41 2 117 124 10.1002/gcc.20062 15287024

[B11] Martinez-Galan J Torres B Del Moral R Munoz-Gamez JA Martin-Oliva D Villalobos M Nunez MI Luna J de D Oliver FJ Ruiz de Almodovar JM Quantitative detection of methylated ESR1 and 14-3-3-sigma gene promoters in serum as candidate biomarkers for diagnosis of breast cancer and evaluation of treatment efficacy Cancer Biol Ther 2008 7 6 958 965 10.4161/cbt.7.6.5966 18379196

[B12] Auwera I Van der Elst HJ Van Laere SJ Maes H Huget P van Dam P Van Marck EA Vermeulen PB Dirix LY The presence of circulating total DNA and methylated genes is associated with circulating tumour cells in blood from breast cancer patients Br J Cancer 2009 100 8 1277 1286 10.1038/sj.bjc.6605013 19367284 PMC2676551

[B13] Baylin SB DNA methylation and gene silencing in cancer Nat Clin Pract Oncol 2005 2 Suppl 1 S4 11 10.1038/ncponc0354 16341240

[B14] Widschwendter M Siegmund KD Muller HM Fiegl H Marth C Muller-Holzner E Jones PA Laird PW Association of breast cancer DNA methylation profiles with hormone receptor status and response to tamoxifen Cancer Res 2004 64 11 3807 3813 10.1158/0008-5472.CAN-03-3852 15172987

[B15] Evron E Umbricht CB Korz D Raman V Loeb DM Niranjan B Buluwela L Weitzman SA Marks J Sukumar S Loss of cyclin D2 expression in the majority of breast cancers is associated with promoter hypermethylation Cancer Res 2001 61 6 2782 2787 11289162

[B16] Evron E Dooley WC Umbricht CB Rosenthal D Sacchi N Gabrielson E Soito AB Hung DT Ljung B Davidson NE Sukumar S Detection of breast cancer cells in ductal lavage fluid by methylation-specific PCR Lancet 2001 357 9265 1335 1336 10.1016/S0140-6736(00)04501-3 11343741

[B17] Fackler MJ McVeigh M Evron E Garrett E Mehrotra J Polyak K Sukumar S Argani P DNA methylation of RASSF1A, HIN-1, RAR-beta, Cyclin D2 and Twist in in situ and invasive lobular breast carcinoma Int J Cancer 2003 107 6 970 975 10.1002/ijc.11508 14601057

[B18] Wu JM Fackler MJ Halushka MK Molavi DW Taylor ME Teo WW Griffin C Fetting J Davidson NE De Marzo AM Hicks JL Chitale D Ladanyi M Sukumar S Argani P Heterogeneity of breast cancer metastases: comparison of therapeutic target expression and promoter methylation between primary tumors and their multifocal metastases Clin Cancer Res 2008 14 7 1938 1946 10.1158/1078-0432.CCR-07-4082 18381931 PMC2965068

[B19] Herman JG Graff JR Myohanen S Nelkin BD Baylin SB Methylation-specific PCR: a novel PCR assay for methylation status of CpG islands Proc Natl Acad Sci USA 1996 93 18 9821 9826 10.1073/pnas.93.18.9821 8790415 PMC38513

[B20] Ferguson AT Evron E Umbricht CB Pandita TK Chan TA Hermeking H Marks JR Lambers AR Futreal PA Stampfer MR Sukumar S High frequency of hypermethylation at the 14-3-3 sigma locus leads to gene silencing in breast cancer Proc Natl Acad Sci USA 2000 97 11 6049 6054 10.1073/pnas.100566997 10811911 PMC18556

[B21] Sasaki M Tanaka Y Perinchery G Dharia A Kotcherguina I Fujimoto S Dahiya R Methylation and inactivation of estrogen, progesterone, and androgen receptors in prostate cancer J Natl Cancer Inst 2002 94 5 384 390 11880477 10.1093/jnci/94.5.384

[B22] Chan TA Glockner S Yi JM Chen W Van Neste L Cope L Herman JG Velculescu V Schuebel KE Ahuja N Baylin SB Convergence of mutation and epigenetic alterations identifies common genes in cancer that predict for poor prognosis PLoS Med 2008 5 5 e114 10.1371/journal.pmed.0050114 18507500 PMC2429944

[B23] Umbricht CB Evron E Gabrielson E Ferguson A Marks J Sukumar S Hypermethylation of 14-3-3 sigma (stratifin) is an early event in breast cancer Oncogene 2001 20 26 3348 3353 10.1038/sj.onc.1204438 11423985

[B24] Jeronimo C Monteiro P Henrique R Dinis-Ribeiro M Costa I Costa VL Filipe L Carvalho AL Hoque MO Pais I Leal C Teixeira MR Sidransky D Quantitative hypermethylation of a small panel of genes augments the diagnostic accuracy in fine-needle aspirate washings of breast lesions Breast Cancer Res Treat 2008 109 1 27 34 10.1007/s10549-007-9620-x 17549626

[B25] Jacinto FV Esteller M Mutator pathways unleashed by epigenetic silencing in human cancer Mutagenesis 2007 22 4 247 253 10.1093/mutage/gem009 17412712

[B26] Wilker EW van Vugt MA Artim SA Huang PH Petersen CP Reinhardt HC Feng Y Sharp PA Sonenberg N White FM Yaffe MB 14-3-3sigma controls mitotic translation to facilitate cytokinesis Nature 2007 446 7133 329 332 10.1038/nature05584 17361185

[B27] Valenzuela MT Mateos S Ruiz de Almodovar JM McMillan TJ Variation in sensitizing effect of caffeine in human tumour cell lines after gamma-irradiation Radiother Oncol 2000 54 3 261 271 10.1016/S0167-8140(99)00180-2 10738085

[B28] Valenzuela MT Guerrero R Nunez MI Ruiz De Almodovar JM Sarker M de Murcia G Oliver FJ PARP-1 modifies the effectiveness of p53-mediated DNA damage response Oncogene 2002 21 7 1108 1116 10.1038/sj.onc.1205169 11850828

[B29] Urano T Saito T Tsukui T Fujita M Hosoi T Muramatsu M Ouchi Y Inoue S Efp targets 14-3-3 sigma for proteolysis and promotes breast tumour growth Nature 2002 417 6891 871 875 10.1038/nature00826 12075357

[B30] Feng W Shen L Wen S Rosen DG Jelinek J Hu X Huan S Huang M Liu J Sahin AA Hunt KK Bast RC Jr Shen Y Issa JP Yu Y Correlation between CpG methylation profiles and hormone receptor status in breast cancers Breast Cancer Res 2007 9 4 R57 10.1186/bcr1762 17764565 PMC2206733

[B31] Dillon RL Brown ST Ling C Shioda T Muller WJ An EGR2/CITED1 transcription factor complex and the 14-3-3sigma tumor suppressor are involved in regulating ErbB2 expression in a transgenic-mouse model of human breast cancer Mol Cell Biol 2007 27 24 8648 8657 10.1128/MCB.00866-07 17938205 PMC2169423

[B32] Niemantsverdriet M Wagner K Visser M Backendorf C Cellular functions of 14-3-3 zeta in apoptosis and cell adhesion emphasize its oncogenic character Oncogene 2008 27 9 1315 1319 10.1038/sj.onc.1210742 17704798

[B33] Wilker EW Grant RA Artim SC Yaffe MB A structural basis for 14-3-3sigma functional specificity J Biol Chem 2005 280 19 18891 18898 10.1074/jbc.M500982200 15731107

[B34] Chu K Teele N Dewey MW Albright N Dewey WC Computerized video time lapse study of cell cycle delay and arrest, mitotic catastrophe, apoptosis and clonogenic survival in irradiated 14-3-3sigma and CDKN1A (p21) knockout cell lines Radiat Res 2004 162 3 270 286 10.1667/RR3221 15332997

[B35] Rajagopalan S Sade RS Townsley FM Fersht AR Mechanistic differences in the transcriptional activation of p53 by 14-3-3 isoforms Nucleic Acids Res 38 3 893 906 10.1093/nar/gkp1041 19933256 PMC2817464

[B36] Schumacher B Mondry J Thiel P Weyand M Ottmann C Structure of the p53 C-terminus bound to 14-3-3: Implications for stabilization of the p53 tetramer FEBS Lett 2010 584 1443 8 10.1016/j.febslet.2010.02.065 20206173

[B37] Iwata N Yamamoto H Sasaki S Itoh F Suzuki H Kikuchi T Kaneto H Iku S Ozeki I Karino Y Frequent hypermethylation of CpG islands and loss of expression of the 14-3-3 sigma gene in human hepatocellular carcinoma Oncogene 2000 19 46 5298 5302 10.1038/sj.onc.1203898 11077447

[B38] Castedo M Perfettini JL Roumier T Yakushijin K Horne D Medema R Kroemer G The cell cycle checkpoint kinase Chk2 is a negative regulator of mitotic catastrophe Oncogene 2004 23 25 4353 4361 10.1038/sj.onc.1207573 15048074

[B39] Sun B Zhang S Zhang D Li Y Zhao X Luo Y Guo Y Identification of metastasis-related proteins and their clinical relevance to triple-negative human breast cancer Clin Cancer Res 2008 14 21 7050 7059 10.1158/1078-0432.CCR-08-0520 18981002

[B40] Rodenhiser DI Epigenetic contributions to cancer metastasis Clin Exp Metastasis 2009 26 1 5 18 10.1007/s10585-008-9166-2 18386135

[B41] Lewis CM Cler LR Bu DW Zochbauer-Muller S Milchgrub S Naftalis EZ Leitch AM Minna JD Euhus DM Promoter hypermethylation in benign breast epithelium in relation to predicted breast cancer risk Clin Cancer Res 2005 11 1 166 172 15671542

[B42] Danes CG Wyszomierski SL Lu J Neal CL Yang W Yu D 14-3-3 zeta down-regulates p53 in mammary epithelial cells and confers luminal filling Cancer Res 2008 68 6 1760 1767 10.1158/0008-5472.CAN-07-3177 18339856

[B43] Neal CL Yao J Yang W Zhou X Nguyen NT Lu J Danes CG Guo H Lan KH Ensor J Hittelman W Hung MC Yu D 14-3-3zeta overexpression defines high risk for breast cancer recurrence and promotes cancer cell survival Cancer Res 2009 69 8 3425 3432 10.1158/0008-5472.CAN-08-2765 19318578 PMC2671640

[B44] Issa JP Age-related epigenetic changes and the immune system Clin Immunol 2003 109 1 103 108 10.1016/S1521-6616(03)00203-1 14585281

[B45] Burzykowski T Buyse M Piccart-Gebhart MJ Sledge G Carmichael J Luck HJ Mackey JR Nabholtz JM Paridaens R Biganzoli L Jassem J Bontenbal M Bonneterre J Chan S Basaran GA Therasse P Evaluation of tumor response, disease control, progression-free survival, and time to progression as potential surrogate end points in metastatic breast cancer J Clin Oncol 2008 26 12 1987 1992 10.1200/JCO.2007.10.8407 18421050

